# The cytomegalovirus gB/MF59 vaccine candidate induces antibodies against an antigenic domain controlling cell-to-cell spread

**DOI:** 10.1038/s41467-023-36683-x

**Published:** 2023-02-23

**Authors:** A. C. Gomes, I. A. Baraniak, A. Lankina, Z. Moulder, P. Holenya, C. Atkinson, G. Tang, T. Mahungu, F. Kern, P. D. Griffiths, M. B. Reeves

**Affiliations:** 1 Institute of Immunity & Transplantation, UCL, London, NW3 2PP United Kingdom; 2 JPT Peptide Technologies GmbH, Berlin, Germany; 3 Experimental Medicine, Brighton and Sussex Medical School, Brighton, United Kingdom

**Keywords:** Infection, Protein vaccines

## Abstract

Vaccination against human cytomegalovirus (CMV) infection remains high priority. A recombinant form of a protein essential for CMV entry, glycoprotein B (gB), demonstrated partial protection in a clinical trial (NCT00299260) when delivered with the MF59 adjuvant. Although the antibody titre against gB correlated with protection poor neutralising responses against the 5 known antigenic domains (AD) of gB were evident. Here, we show that vaccination of CMV seronegative patients induces an antibody response against a region of gB we term AD-6. Responses to the polypeptide AD-6 are detected in >70% of vaccine recipients yet in <5% of naturally infected people. An AD-6 antibody binds to gB and to infected cells but not the virion directly. Consistent with this, the AD-6 antibody is non-neutralising but, instead, prevents cell-cell spread of CMV in vitro. The discovery of AD-6 responses has the potential to explain part of the protection mediated by gB vaccines against CMV following transplantation.

## Abstract (web summary)

A gB/MF59 vaccine candidate for human cytomegalovirus (HCMV) provided partial protection in organ transplant patients. Here, the authors identify antibody responses in trial participants that target virally infected cells to control cell-to-cell spread of HCMV, providing a potential mechanism for the observed protection.

## Introduction

Human cytomegalovirus (CMV) is a highly prevalent (65–95%) herpesvirus that causes limited disease in the immunocompetent^1, 2^. In contrast, in the presence of an immunosuppressed or immature immune system, CMV infection is a major cause of morbidity and mortality—it is the leading viral cause of congenital disease^3^ and a major complication post-organ transplant^4, 5^. The economic and health costs associated with infection mean the development of a vaccine against CMV has been considered the highest priority^6, 7^.

CMV has a broad cell tropism and can utilise multiple entry pathways^8, 9^. Furthermore, CMV can infect cells via cell-free and cell-associated mechanisms^8, 10^. An essential component of CMV entry is glycoprotein B (gB) a 907-amino acid long protein that forms a homo-trimeric complex on the surface of the virion^10^. gB contains three topological domains: an ectodomain, a transmembrane domain, and the cytoplasmic (or intraviral) domain^11^. Importantly, gB is an immunodominant protein and an important target of neutralising antibodies. The humoral response against gB following natural infection is largely directed against five antigenic domains (named AD-1–5) with different immunodominance and distinct contribution to neutralising responses^12^.

Considering the key role of gB in HCMV infection and immunity, several vaccine candidates included gB as the antigen of choice^13^. Of the vaccine candidates tested in humans, the gB/MF59 vaccine containing soluble recombinant gB with the MF59 adjuvant has performed the best in phase II clinical trials achieving 43–50% efficacy in three different clinical trials^14–16^. Although the total gB antibody titre is a correlate of protection^17^ the exact mechanism remains unknown. Our research has focused on sera taken from a phase II randomised trial performed in solid organ transplant patients^15^. We previously reported that the protective effect of this vaccine is not via induction of neutralising antibodies^18^. Furthermore, ELISA analyses did not detect substantial responses against AD-1–5 of gB suggesting that vaccination induced an atypical humoral response compared to natural infection^18^.

Here we now show that vaccination with gB/MF59 induces a strong humoral response directed against a region of the gB protein we are calling AD-6. Responses against this region are rarely detected in naturally infected individuals. Rabbit antibodies directed against AD-6 are non-neutralising but bind to infected cells preventing cell-to-cell spread of HCMV. Importantly, we identify sera from vaccine recipients that can limit cell-to-cell spread and demonstrate that this is dependent on AD-6 antibodies. Taken together, the data suggest an atypical response induced by vaccination could explain the protection observed with gB/MF59 vaccine in transplant patients.

## Results

**Fig. 1:**
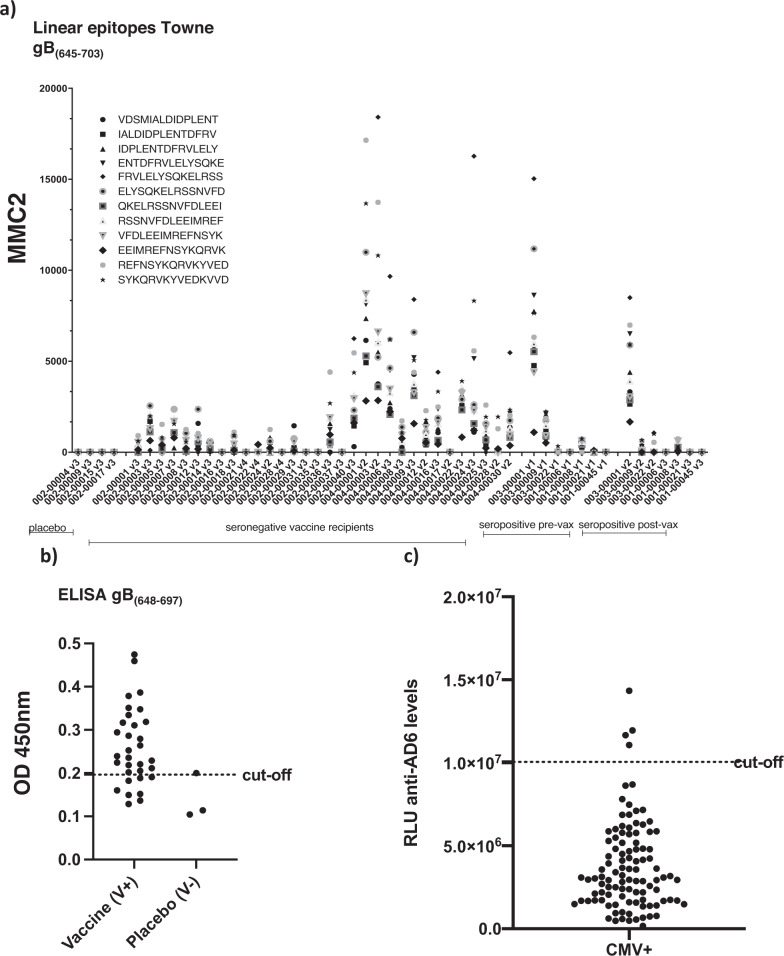
Seronegative gB/MF59 recipients develop humoral responses against novel epitopes in gB. **a** IgG levels against 15-mer peptides spanning gB_(645–703)_ (Towne) in seronegative vaccine recipients after the second dose of the vaccine. Microarrays were scanned using a high-resolution fluorescence scanner. For each spot, mean signal intensity was extracted (between 0 and 65535 arbitrary units). The MMC2 equals the mean value of all three instances on the microarray. Each peptide is represented by the symbol in the key (**b**) Anti-AD-6 levels (gB_(648–697)_ Towne) in 32 seronegative recipients of vaccine (V+) or 3 placebo (V−) measured by ELISA and represented as absorbance at 450 mm. Cutoff calculated as average of 2 seronegative sera plus 4 standard deviations. **c** Anti-AD-6 IgG (gB_(648–697)_ Towne) levels in 102 CMV seropositive volunteers measured by chemiluminescence immunoassay (CLIA). Cutoff was determined by averaging values of seven sera samples from CMV seronegative volunteers plus 4 standard deviations. Values are expressed as relative light units (RLU).

The detection of high titres of gB antibodies post vaccination coupled with a distinct lack of responses against known ADs of gB led us to hypothesise that vaccination induced responses against a novel AD. To investigate this, we performed linear epitope mapping studies of gB in CMV seronegative gB vaccine recipients. Antibodies against linear epitopes were screened for against an array comprising a 15-mer peptide library covering the entire gB open reading frame revealing a distinct pattern of response compared to CMV seropositive individuals (Figure S1). We noted that strong responses were directed against the intraviral/intracellular domain of gB AD-3 (Figure S1) and also against a number of epitopes that overlapped with Domain V (Fig. 1a) with a number of strong responses directed against peptides sitting within Domain V itself (aa 648–697 in Towne)^11^. Thus we decided to investigate responses directed against epitopes within Domain V further. Using an ELISA approach, 78% (25 out of 32) of seronegative patients receiving vaccination had a detectable IgG response against an in vitro synthesised polypeptide mapping to a region within gB corresponding to the amino acids in position 648 to 697 (Towne strain) in the carboxy-terminal ectodomain of the protein (Fig. 1b). In stark contrast, less than 5% of naturally infected individuals had detectable responses against epitopes within this region – even when a more sensitive assay was used (Fig. 1c). Thus we termed this region antigenic domain 6 (AD-6).

**Fig. 2:**
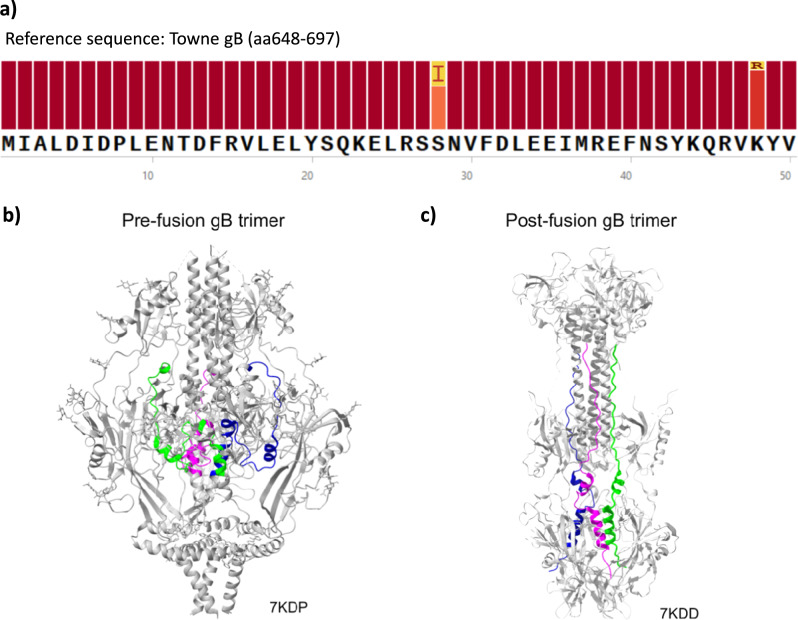
AD-6 sequence conservation and location in gB trimer. **a** A multiple sequence alignment of gB sequences from Genbank was assembled using MAFFT online server. Sequences from synthetic strains were removed. The Towne strain (GenBank: ABQ23592.1) was used as a reference. **b**, **c** The three AD-6 regions (gB_648-697,_ Towne) are highlighted in green, blue and pink in the gB trimer represented by the pre-fusion (PDB: 7KDP **b**) and post-fusion (PDB: 7KDD **c**) state.

**Fig. 3:**
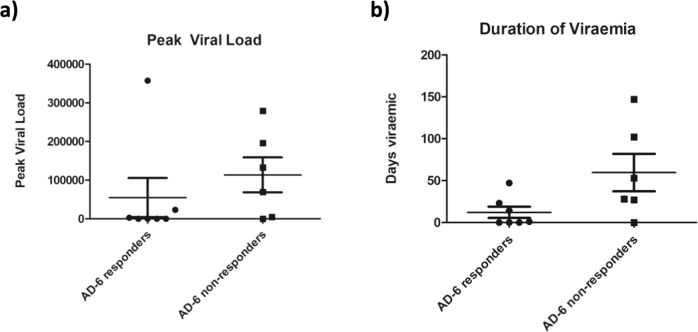
Seronegative vaccine recipients receiving an organ from a seropositive donor have reduced viral loads and duration of viraemia post-transplant. **a** Peak viral load post-transplant in D + R- AD-6 responders (*n* = 6) and non-responders (*n* = 7). **b** Duration of viremia post-transplant in D + R- AD-6 responders (*n* = 7) and non-responders (*n* = 6). *N* = number of patients in each cohort and the mean plus 1 standard deviation from the mean are shown.

A sequence alignment analysis of AD-6 (retrieved from 390 genomes deposited in the NCBI database) reported the region to be nearly completely conserved at the amino acid level across compared sequences with 96.1% of identical sites, 99.98% of pairwise identity, and two substitutions observed at S28I and K48R which correspond to residues 675 and 695 in Towne gB (Fig. 2a). The high degree of conservation observed may be a consequence of the essential role of the region in the fusogenic machinery of gB. The recently published structure of gB in the prefusion state revealed the structural basis of fusion^19^, demonstrating the extensive refolding of gB around aa 639–704 (Towne gB) that happens in the transition to the pre-fusion to post-fusion state. Given the overlap between AD-6 and Domain V (and it being part of the larger domain aa 639–704) we modelled AD-6 onto the pre-fusion structure which demonstrates that AD-6 is similarly buried within the trimer (Fig. 2b). During structural re-arrangement into the post-fusion form, the prefusion alpha helices become extended coils, which led to AD-6 being presented externally on the trimeric structure in silico (Fig. 2c). Taken together, these data pointed towards the presence of an immune response against specific epitopes of gB observed in vaccinated patients - which here we have termed AD-6 – that was less common during natural infection which may be due to limited exposure of AD-6 epitopes in natural infection compared to the vaccine. Crucially, a retrospective analysis of the original gB vaccine study^15^ suggested that seronegative vaccine recipients who proceeded to transplant had better clinical outcomes if they had also developed an AD-6 response upon vaccination and prior to transplantation (Fig. 3).

Polypeptides containing gB-like heptad repeat motifs – including those derived from sequences overlapping Domain V (e.g. aa 675–703 in AD169)—have been shown to inhibit infection of fibroblasts with laboratory-adapted strains of CMV in a dose-dependent manner^20^. Here we show that our AD-6 polypeptide (aa 648–697 Towne) also inhibited infection of fibroblasts with the clinical isolate Merlin but, intriguingly, they did not prevent epithelial cell infection (Figure S2). A + 4°C binding assay suggested that AD-6 polypeptide did not block virus binding to the cell surface (Figure S3A). Consistent with this, AD-6 polypeptide had no impact on known gB-mediated induction of interferon-stimulated genes (ISG) that occurs post-attachment of CMV^20^ (Figure S3B). Taken together, these data argue that the polypeptide has post-attachment impact on CMV infection in HFFs with clear similarities to the findings reported by Lopper et al.^20^

**Fig. 4:**
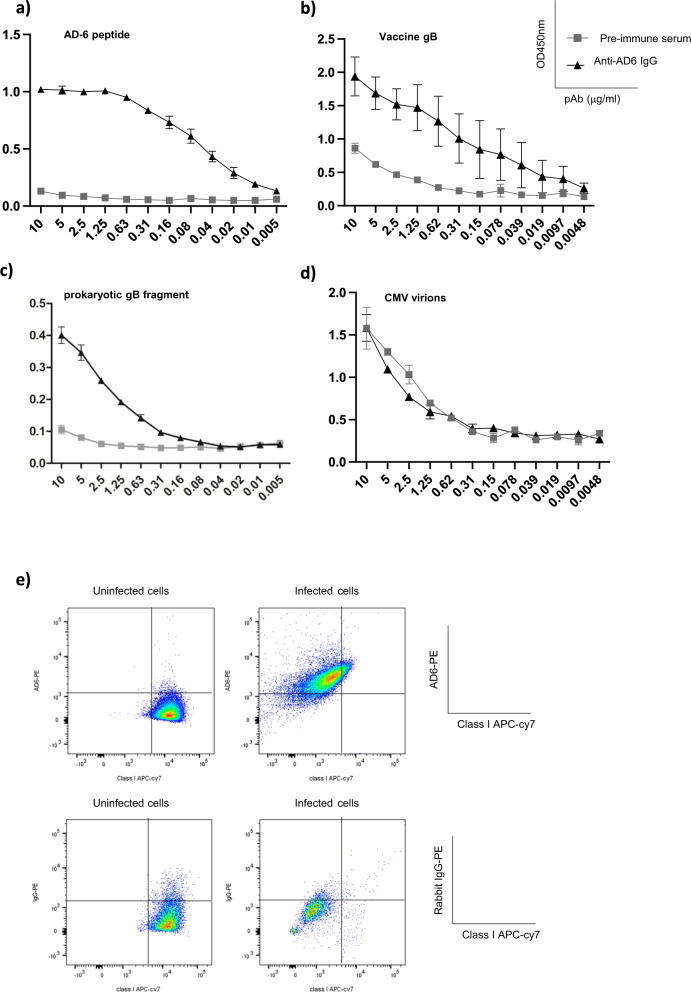
AD-6 antibodies recognise gB vaccine, recombinant gB and infected cells but not virions. **a**–**d** An anti-AD-6 polyclonal antibody was assayed by ELISA for binding to AD-6 polypeptide (**a**), vaccine gB (**b**), a commercial recombinant gB produced in *E. coli (***c**) or CMV virions (**d**). Antibody levels are represented in function of absorbance (450 nm) per concentration of primary antibody in μg/mL and is representative of three independent experiments. Each data point represents mean + SEM of antibodies purified from two immunized rabbits. **e** Flow cytometry analysis of mock-infected and CMV-infected cells with anti-AD-6 pAb or non-specific rabbit IgG 3 days post infection. Gated on single, live cells and HLA class I expression to identify virally infected cells.

The inhibitory effect of the AD-6 polypeptide on infection led us to investigate whether antibodies directed against AD-6 could neutralise cell-free virus. To do this, we raised a polyclonal rabbit serum against the AD-6 polypeptide (aa 648–687 Towne)—and then affinity-purified against AD-6 to obtain an AD-6 polyclonal antibody. We confirmed specificity of the anti-AD-6 IgG polyclonal antibody (pAb) against AD-6 (Fig. 4a) and furthermore, we demonstrated that the anti-AD-6 pAb recognised the modified version of gB used in the gB/MF59 vaccine (Fig. 4b). Next, we assessed the capacity of the AD-6 pAb to bind to other forms of gB. The antibody bound a recombinant gB fragment that had been produced in bacteria (Fig. 4c) albeit it appeared less efficiently than the modified form of gB used in the vaccine which had been made in Chinese hamster ovary cells^12^ (Fig. 4b). In contrast, the binding of an anti-AD-2 monoclonal antibody (ITC88)^21^ was similar for both the recombinant gB fragment and vaccine gB but did not bind to the AD-6 polypeptide (Fig. S4A–C). Furthermore, we could find no evidence of specific AD-6 pAb binding directly to CMV virions (Fig. 2d) in contrast to ITC88 which, consistent with its role as a neutralising antibody^21^, strongly bound virions (Figure S4D). Clearly, the data suggested that the AD-6 pAb may only bind gB in certain circumstances. Thus we decided to test whether AD-6 pAb could recognise gB at the plasma membrane in the context of infection. To do this, cells were infected with Merlin (MOI:5) and then incubated with AD-6 pAb and analysed by FACS. Infected cells were identified using the surrogate marker of MHC class I down-regulation to avoid the need to permeabilise the cells which proved problematic when using the unconjugated rabbit AD-6 pAb antibody. Using this approach, we observed the majority of cells displayed reduced MHC class I expression and this was concomitant with evidence of AD-6 pAb binding to the surface of the cells suggesting the AD-6 pAb can recognise and bind gB on the cell surface (Fig. 4e).

**Fig. 5:**
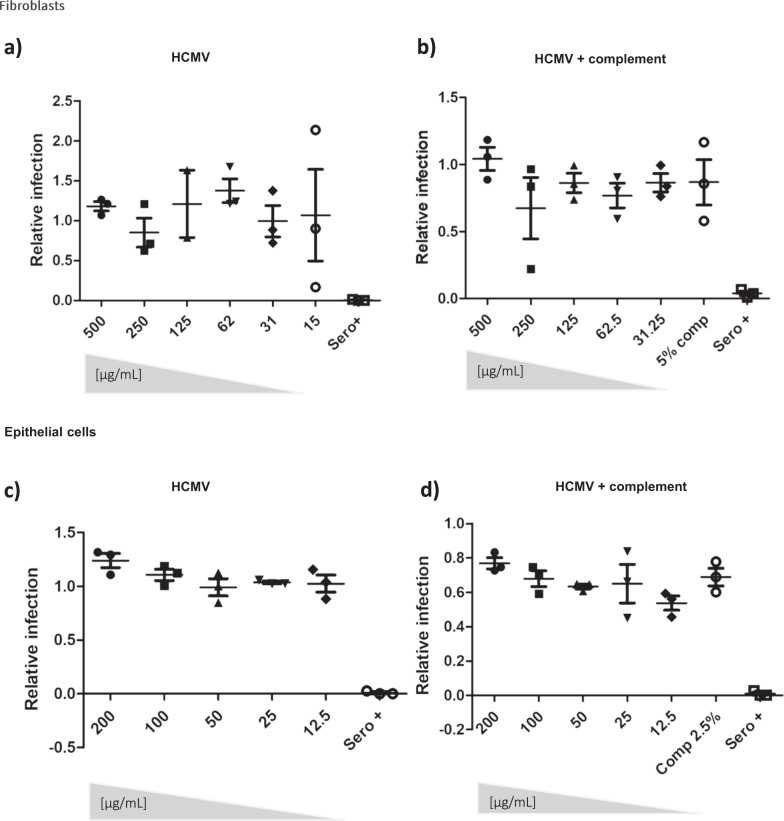
An Anti-AD-6 antibody is non-neutralising against cell-free CMV. **a**, **b** Serial dilutions of anti-AD-6 pAb were incubated with Merlin (**a**), Merlin in the presence of complement (**b**) for 1 hour then used to infect HFFs (MOI = 1) and infection scored by IF for IE gene expression. Infection was expressed relative to infection in a no antibody control. **c**, **d** Serial dilutions of anti-AD-6 pAb were incubated with TB40/E (**c**), TB40/E in the presence of complement (**d**) for 1 hour then used to infect ARPE-19 cells (MOI = 2) and infection scored by IF for IE gene expression. Infection was expressed relative to infection with a no antibody control. For all experiments (**a**–**d**) *n* = 3 represents the mean of three independent experiments which included biological triplicates within each experiment. The error bars represent 1 standard deviation from the mean.

Although these phenotypic and clinical data were important they did not demonstrate any functional anti-viral activity of humoral responses against epitopes within AD-6. Thus to investigate whether the AD-6 pAb had any direct anti-viral activity we first investigated the ability to limit infection of fibroblasts and epithelial cells (Fig. 5). The data show that the AD-6 pAb displayed negligible neutralising activity against cell-free CMV (Fig. 5a, c) even in the presence of complement (Fig. 5b, d). A lack of neutralising activity is consistent with our previous studies of pre-transplant vaccine recipients’ sera which could find no evidence of neutralising activity^18^. However, we have also reported that seronegative vaccine recipients who then proceed to transplant do display detectable neutralising anti-gB responses in the post-transplant phase after exposure to a transplanted allograft containing CMV^22^. Thus, we next asked whether AD-6- specific antibodies were a component of the gB-specific neutralising response observed post-transplant in those patients. An analysis of neutralising activity post-transplant in relation to IgG gB_(648-697)_ levels in our cohort of D^+^R^-^ patients revealed there was no correlation between evidence of neutralisation and an AD-6 response suggesting other gB humoral responses were important for the post-transplant neutralising activity we observed (Figure S5).

**Fig. 6:**
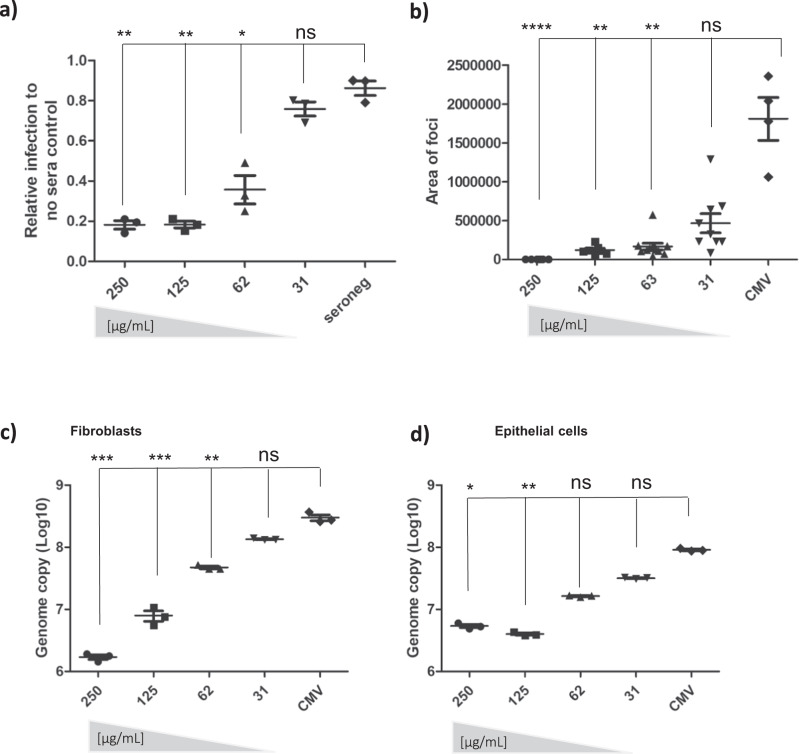
Anti-AD-6 antibodies limit cell-associated spread of CMV. **a**–**c** To measure spread of cell-associated virus in HFFs, cells were infected with Merlin-IE2-GFP (MOI = 0.01). At 1 dpi and 5 dpi, anti-AD-6 antibody was added at the indicated concentrations (plus control normal rabbit sera in A. At 10 dpi, cells were then analysed by IF or DNA-qPCR for viral spread. Percentage of infected cells (**a**) was measured by anti-IE stain counterstained with nuclei stain and counted by automated fluorescence microscopy (*n* =  3) and expressed relative to infection seen in cells incubated with no sera. Alternatively, the area of each individual plaque identified from randomly chosen images from the three independent experiments analysed in **a** was measured using Fiji software (**b**). Alternatively, total DNA was harvested, and CMV genome copies per 10^6^ cells assessed by qPCR (**c**; *n* = 3 independent experiments reporting on biological replicates). **d** ARPE-19 cells were infected with pentamer positive BAC derived Merlin-IE2-GFP (MOI = 0.01), and total DNA harvested 20 dpi and analysed for viral genome copy number per 10^6^ cells *n* = 3; independent experiments reporting on biological replicates. For all analyses (**a**–**d**) *P* values were calculated by a Kruskal–Wallis test with Dunn’s multiple-comparison test where appropriate. ****P < 0.0001; ***P < 0.001; **P < 0.01 *P < 0.05; ns *P* > 0.05. In all cases (**a**–**d**), the error bars represent 1 standard deviation from the mean.

The data suggesting antibody binding to gB on the surface of infected cells led us to hypothesise that, if true, AD-6 antibodies could potentially mediate anti-viral effects against cell-to-cell spread of CMV—arguably the major route of CMV infection within a host. To test this, we used a strain of CMV engineered to grow highly cell-associated^23^. Fibroblasts were infected at low MOI infection monitored over 10 days and then quantified by immunostaining for viral infection, plaque size, and viral genome copy number. The data show that the addition of the AD-6 antibody at one and then again at five days post infection dramatically reduced viral spread and plaque size in a dose-dependent manner (Fig. 6a, b). Additionally, a viral genome copy assay demonstrated a ~2 log reduction in the viral load 10 dpi in the presence of the highest concentration of antibody tested in our assay (Fig. 6c). Importantly, this effect was not fibroblast-specific - the antibody also reduced viral spread in epithelial cells where HCMV also grows highly cell-associated (Fig. 6d). Finally, using a short peptide from AD-6 (AD-6.4; aa 687–701 Towne) against which a number of patients made high responses (Fig. 1a) we found that pre-absorption of the AD-6 pAb with this peptide partially reduced the capacity of the pAb to control the virus suggesting that a component of the polyclonal response against AD-6 could involve this epitope (Figure S6).

**Fig. 7:**
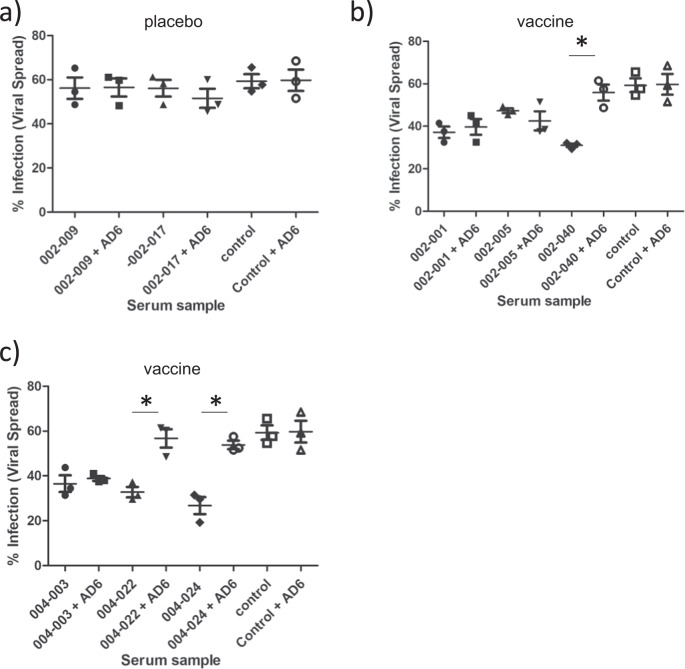
The AD-6 polypeptide reverses the control of HCMV spread by human sera taken from gB vaccine recipients. **a**–**c** To measure the spread of cell-associated virus in HFFs, cells were infected with Merlin-IE2-GFP (MOI = 0.01). At 1 dpi and 5 dpi, healthy seronegative sera (control), or sera from placebo (**a**) or gB vaccinated (**b**, **c**) individuals was added at a 1:20 dilution. Additionally, sera were also pre-incubated with AD-6 peptide prior to addition to the infected cells (+AD-6). 10 dpi, cells were then analysed by IF for viral spread. The percentage of infected cells was measured by anti-IE stain counterstained with nuclei stain and counted by automated fluorescence microscopy (*n* = 3 where each point is the average of a biological duplicate that was performed for three independent experiments). *P* values were calculated by a two-sided Mann-*U*-Whitney comparison of means test. *p = 0.00512; where a *p* value is not indicated differences were non-significant. The error bars represent 1 standard deviation from the mean.

Finally, we wanted to determine whether we could observe any equivalent anti-viral activity associated with AD-6 antibodies in sera from recipients of the gB vaccine. Although previously we have reported that vaccine recipient sera did not have a dramatic impact on viral spread^18^ we noted two caveats: firstly, the sera analysed were at the time of transplant and thus not when gB antibody titres were highest and secondly, inspection of the individual patient data^18^ suggested that some sera displayed evidence of control. Furthermore, we noted that many of these patients with evidence of control also had detectable AD-6 responses. Thus, we performed a re-analysis of these patients’ sera using the sample with highest gB antibody titre (clinic visit 3 which is 1 month after second dose of MF59/gB vaccine^15^). Using our standard spread assay, sera were added to infected HFFs and then after 1 day and then at 5 days sera from visit 3 or sera plus AD-6 peptide were added. The spread of the virus was assayed at day 10 and as expected sera from seronegative patients who received placebo did not control spread of HCMV and thus no effect of adding AD-6 peptide was observed (Fig. 7a). In contrast, sera from vaccinated individuals awaiting kidney (Fig. 7b) or liver (Fig. 7c) displayed evidence of control of HCMV spread in these assays. Intriguingly, for some patient sera this control was reversed following addition of AD-6 peptide (Fig. 7b, c, e.g., 002-0040; 004-022; 004-0024) replicating the observations made with the rabbit polyclonal AD-6 antibody in the sera of vaccine recipients.

## Discussion

In this study of stored sera from a placebo-controlled randomised phase II trial, we have made the exciting discovery of a novel vaccine-directed antibody response against epitopes in a region of gB (AD-6). Interestingly, responses against this region were rarely detected in naturally infected individuals but enriched in HCMV seronegative individuals receiving the gB/MF59 vaccine. These differences in the humoral repertoire may be explained by differences in the tertiary and/or quaternary structure of gB used in the vaccines and native gB. Similarly, to other type III viral fusion proteins such as VSV-G^24^, gB-mediated fusion in herpesvirus occurs following substantial structural re-arrangement^19^. As such, native gB exists in both the pre-fusion and post-fusion conformations^19,25^. In general, for a given protein in different conformational states, distinct binding sites would be available for B cell binding, activation, and selection resulting in a unique antibody repertoire and, indeed, using well-defined crystal structures of gB we can observe how, theoretically, AD-6 can be internal (pre-fusion) and external (post-fusion). To understand the basis of why the gB/MF59 vaccine was particularly good at inducing AD-6 responses it is necessary to consider its original design over 30 years ago. Often when soluble proteins are used as antigens, further modifications, and truncations are introduced. Depending on the type of modifications and even the protein expression system chosen for production, the protein may present distinct conformational or glycosylation patterns. The Chiron gB used in the gB/MF59 vaccine, is a truncated version of gB lacking the transmembrane domain^14^. Consequently, the immunogen included in the vaccine may serendipitously expose regions that are not efficiently presented by the virion during natural infection. This has been shown to be true for gB, where extensive changes in the antigenic map are observed in the pre and post-fusion forms^19^. The observation that AD-6 antibody did not strongly and specifically recognise the virion—in which gB is predominantly in the pre-fusion form (~79%)^25^—is supportive of this. This lack of reactivity with virion gB likely explains why the antibody failed to neutralise infection and is also consistent with previous work that demonstrated the antibody response to vaccine gB was largely non-neutralising^18,26^ which of course is the same sera^18^ used to identify AD-6.

Although differences in vaccine versus native gB structure may explain differences in the antibody repertoire it does not completely explain why if AD-6 can be recognized in the context of infected cells by antibodies we rarely see these antibodies in vivo. We know from studies of AD-2 that the VDJ re-arrangement required is a rare event^27,28^. However, we and others still see 50% AD-2 reactivity in HCMV seropositive individuals^29–31^—much higher than the 5% we report here for AD-6. One possibility is that gB predominantly adopts a trimeric conformation in vivo and thus the exposure of AD-6 is largely limited in natural infection both in the virion and at the plasma membrane. However, we cannot rule out the presence of gB monomers in the vaccine preparation which may have presented AD-6 epitopes—against which effective antibodies might be made. If the AD-6 region is important for gB function, and is intolerant to mutation, then reducing the exposure of this region from humoral immunity makes evolutionary sense as an immune evasion strategy. However, the observation that AD-6 antibodies do bind to infected cells suggests that AD-6 is exposed transiently during certain phases of the viral infection cycle. A caveat of this is that in the FACS analysis a spectrum of AD-6 staining of infected cells was observed which may suggest gB is only recognised by the AD-6 pAb for a limited time. Future investigations will look to see if there is an optimum time in the infection cycle when the AD-6 domain of gB is most exposed at the plasma membrane. Indeed, transient exposure at the plasma membrane during infection would again explain the poor induction of de novo responses against AD-6 in natural infection. Evasion of humoral immunity directed against important regions of viral fusion proteins usually encompasses one of two main strategies: antigenic variation or antigen masking. Given the lack of sequence divergence in this region of gB we propose the latter explanation most easily explains the data.

Crucially, AD-6 can be recognised in the context of CMV infection—specifically, the antibodybound to gB at the surface of infected cells. Importantly, this binding translated into a reduction of viral replication and spread by the cell-to-cell route. Cell-associated spread is considered a viral mechanism to evade antibody neutralisation and has been reported for HIV^32^ and CMV^33^. Indeed, strains of laboratory-adapted CMV have acquired mutations that increase cell-free production; when these viruses are genetically repaired to represent the clinical sequence, they become highly cell-associated^23^. We hypothesise that blockade of gB by the antibody may limit the capacity of infected cells to make contact with neighbouring cells—it also suggests a mechanism by which a vaccine that did not induce neutralising antibodies^18, 26^ was still able to demonstrate partial efficacy in vivo^14–16^. Indeed, a study with a mouse model of transplantation suggested that non-neutralising antibodies protected against CMV disease^34^. Recently, Jenks et al. provided evidence that antibody binding to gB presented on the surface of infected cells, but not binding to soluble vaccine antigen, was also associated with a decreased risk of CMV acquisition in seronegative women vaccinated with the same gB/MF59 vaccine^35^. Thus, binding to the cell-associated gB was found to be a humoral correlate of protection. Here, we demonstrate epitopes within gB that are available in the context of CMV-infected cells both in fibroblasts and epithelial cells, is recognised by antibodies directed against it, and that those antibodies are functional in that they reduce the cell-cell spread of this virus. The observation that sera from some vaccine recipients could limit cell-to-cell spread and that this was reversed with AD-6 peptides also suggests that human AD-6 antibodies induced in response to vaccination can also control HCMV and possibly partially explains the protection observed with this vaccine in, at least, the transplant patient population. We also note that in some cases there were sera that controlled cell-to-cell spread of the virus but could not necessarily be explained by AD-6 in our assay conditions. Since these individuals only had gB antibody responses (since these were pre-transplant sera taken from HCMV seronegative individuals vaccinated with gB) it suggests that other gB antibody responses induced by the vaccine could also be important^17, 36^. Work is ongoing to try and identify them but we highlight, for example, recent work that demonstrates an anti-AD-5 antibody that is a potent inhibitor of gB fusogenic activity which could contribute to the limiting of viral cell-to-cell spread^37,38^. Indeed, there is a certain hubris to imply a single immune response is required for complete protection from HCMV infection – the key will be identifying the important responses and finding ways to induce them effectively whilst eliminating the induction of poorer, competing responses.

In summary, we have identified and characterised anantigenic domain in CMV gB vaccine recipients rarely observed in naturally infected people. Characterisation of an AD-6 antibody generated in rabbits shows that responses against this epitope are non-neutralising but limit the spread of cell-associated CMV. These mechanistic data, paired with the retrospective analysis suggesting that AD-6 responses correlated with better outcomes post-transplant and also demonstrated control of HCMV in vitro, provide strong evidence that AD-6 (and the immune responses raised against it) represent a potential correlate of immune protection that should become a key component of future CMV vaccine studies. Finally, we propose that future vaccination strategies against HCMV should focus on generating potent antibody responses against epitopes that promote control of cell-associated and cell-free virus as this will be important for the control of dissemination and transmission of HCMV. Furthermore, it could serve as an exemplar of a general vaccine strategy for the control of other chronic viral infections.

## Methods

### Ethics statement

The follow-up study was approved by the UCL Research Ethics Committee and all patients whose samples were investigated here gave written informed consent.

### Patient population

The studies comprised a subset of a cohort of solid organ transplant patients enroled in a phase 2 randomised, double-blind, placebo-controlled CMV gB vaccine with MF59 adjuvant trial registered with ClinicalTrials.gov and gave consent for inclusion in trial and follow-up studies (identifier NCT00299260)^15^. Selection of the subset cohort analysed by array was based on whether patients were HCMV seronegative prior to vaccination with gB/MF59. For specific studies of viraemia and neutralisation in patients who proceeded to transplant selection criteria focused on criteria where CMV status of recipient (CMV negative, R−), and organ transplanted (donor CMV seropositive, D+) were confirmed.

### Cell culture

Human retinal pigment epithelial (ARPE-19; ATCC: CRL-2302) cells were maintained in Dulbecco’s modified Eagle medium-12 (DMEM-F12) supplemented with 10% FCS, 2mM L-glutamine, 1 mM sodium pyruvate, 50 U/mL penicillin, 50 μg/mL streptomycin.

Human Foreskin Fibroblast (HFF) (ATCC: SCRC-1041) cells were maintained in Dulbecco’s modified Eagle medium (DMEM) supplemented with 10% FCS, 2mM L-glutamine, 50 U/mL penicillin, 50 μg/mL streptomycin.

CMV Merlin, BAC-derived IE2-GFP, and TB40/E-UL32-GFP stocks of CMV were propagated in HFFs and were kind gifts of Richard Stanton (Cardiff University: Merlin) and Christian Sinzger (Ulm University: TB40/E-UL32-GFP).

### gB Peptide array

As described in detail^39^. Briefly, to identify linear gB epitope binding, 224 15-mer peptides covering the entire gB open-reading frame (Towne strain, UniProt ID: P13201, GB_HCMVT) and overlapping with neighbouring peptides by 5aa each side were synthesised and printed to a PepStar multi-well array (JPT Peptide) in triplicate. Human and mouse IgG printed to the array as assay controls. Sera was diluted 1:200 in 50 mM TBS buffer plus 0.1% Tween 20 and incubated for 1 h on arrays at 30 °C. After washing, bound antibodies were detected using Anti-human-IgG (H + L)-Alexafluor 647 (Jackson Immunoresearch 109-605-098; 0.1 μg/ml) incubated with arrays for 1 hr. Arrays were scanned using Axon Genepix Scanner 4300 SL50 and then quantified using spot-recognition software GenePix (Molecular Devices). For each spot, mean signal intensity was extracted (between 0 and 65535 arbitrary units). For further data evaluation, the so-called MMC2 values were determined. The MMC2 equals the mean value of all three instances on the microarray, except when the coefficient of variation (CV) – standard deviation divided by the mean value—is larger 0.5. In this case, the mean of the two closest values (MC2) is assigned to MMC2.

### In Silico analysis of AD-6 sequence and gB structure

In order to perform sequence conservation analysis, a selection of 424 full-length amino acid sequences were downloaded from GenBank (search terms: “Human betaherpesvirus 5 glycoprotein B “, filtered by 880-920 amino acid sequence length). A multiple sequence alignment was assembled using MAFFT online server. (10.1093/bib/bbx108). Sequences from synthetic strains were removed. Towne strain (GenBank: ABQ23592.1) was used as a reference. To study AD-6 location in the gB structure the region corresponding to AD-6 (aa 648 – 697, Towne) were highlighted in each side chain of the Homotrimer of Towne Glycoprotein B structure in the prefusion (PDB: 7KDP) and post-fusion (PDB: 7KDD) conformation. Structure files were collected from https://www.rcsb.org/. Visualisation was performed using 3-D structure viewer Geneious Prime® 2021.2.2

### Peptides and antibodies

Custom-made AD-6 (CMIALDIDPLENTDFRVLELYSQKELRSSNVFDLEEIMREFNSYKQRVKYV with a GGC tag) and AD-6.4 (REFNSYKQRVKYVED with a GGC tag) were purchased from Peptide 2.0 US. Custom-made affinity-purified anti-AD-6 rabbit IgG was generated by GenScript.

A Recombinant gB fragment was purchased (Abcam; ab43040) and the vaccine gB was a kind gift of Sanofi Pasteur.

Anti-IE CMV (6F8.2, MAB8131, Millipore; 1:2000 dilution). Goat anti-mouse IgG (H + L)-AlexaFluor 568 (A11004, Invitrogen, 1:2000 dilution) and Live/Dead Aqua viability stain (Life Technologies, 1:1000 dilution). The ITC88 antibody was purchased from CreativeLabs.

### Antibody-mediated neutralisation assays and peptide inhibition assay

Human Foreskin Fibroblast (HFF) cells and Human retinal pigment epithelial (ARPE-19) were seeded at a density of 10^4^ cells per well in a 96-well cell culture plate in complete Dulbecco’s Modified Eagle Medium (cDMEM) for HFF and DMEM-F12 for ARPE-19.

To assess antibody-mediated neutralisation, CMV was pre-incubated with heat-inactivated serum samples (dilution of 1:10) for 1 h at 37 °C prior to infection. To assess peptide inhibition of infection, CMV was pre-incubated with AD-6 peptide prior to infection of cells. To assess complement-dependent antibody function, identical experimental conditions were used with addition of 2.5–5% guinea pig complement (SIGMA) to the sera:virus mix prior to infection.

For pre-absorption experiments, anti-AD-6 antibody was pre-incubated with 10× molar excess of AD-6 or AD-6.4 peptide for 1 h at 37 °C prior to incubation with virus.

In all cases, cells were fixed 24 h post infection by treatment with 100% ice-cold ethanol for >20 min at –20 °C. Cells were stained for expression of viral immediate early (IE) protein expression and nuclei counterstained with 0.5 μg/ml DAPI (4′,6-diamidino-2-phenylindole) for 1 h. (Sigma). Percentage infection was assessed by automated fluorescence microscopy and image recognition Hermes WiScan (IDEA Bio-Medical) instruments and processed by MetaMorph software (Molecular devices).

### Viral spread assay

HFF or ARPE-19 cells were infected with IE2-GFP virus engineered to grow predominantly cell-associated^23^ at an MOI of 0.01. Anti-AD-6 antibody was added to infected cells 1 dpi and 5 dpi, and media and treatments were replenished every 5 days. Cells were fixed from 10 to 20 dpi and stained for IE expression or lysed for viral DNA extraction and CMV genomes copy measured by qPCR.

### Nucleic acid extraction

DNA was extracted by proteinase K digestion of infected cells using DNA extraction Qiagen kit according to the manufacturer’s instructions. DNA was eluted from the Qiagen columns in a final volume of 50 μl of distilled water and was stored at −70 °C until used. DNA samples were used for both qualitative and quantitative PCR assays.

RNA was extracted using a Qiagen RNeasy kit, with columns from Epoch Life Sciences, according to the manufacturer’s instructions. cDNA was then synthesised from 250 ng total RNA using a Qiagen Quantitect reverse transcription kit according to the manufacturer’s instructions.

### Quantitative real-time PCR

Absolute quantification by qPCR for viral genome replication was performed using PowerUp SYBR green master mix (ThermoFisher) as per manufacturer instructions. Forward and reverse primers (100 nM) corresponded to nucleotides 1942 to 1964 and 2066 to 2031 of the CMV gB ORF(F, GAGGACAACGAAATCCTGTTGGGGA; R, TCGACGGTGGAGATACTGCTGAGG). A quantitative standard curve was built using seven dilutions of a plasmid standard containing the UL55 gene (gB) of CMV.

Relative quantification by qPCR was performed using PowerUp SYBR green master mix (ThermoFisher) with forward and reverse primers (100 nM) according to the manufacturer’s instructions, using an Applied Biosystems 7500 real-time PCR system (50 °C for 2 min; 95 °C for 2 min; 40 cycles of 95 °C for 15 s, 60 °C for 15 s, and 72 °C for 1 min). The data were analysed by the ΔΔ*C*_*T*_ method using 18 S RNA as a housekeeping gene. The following gene-specific primers (Invitrogen) were used: 18 S (F, GTA ACC CGT TGA ACC CCA; R, CCA TCC AAT CGG TAG TAG CG), UL138 (F, GAG CTG TAC GGG GAG TAC GA; R, AGC TGC ACT GGG AAG ACA CT), IFIT2 (F, ACT GCT GAA AGG GAG CTG AA; R, TGC ACA TTG TGG CTT TGA AT), IFIT3 (F, AGA AAT GAA AGG GCG AAG GT; R, ATG GCC TGC TTC AAA ACA TC), and CXCL10 (F, TGG CAT TCA AGG AGT ACC TC; R, TTG TAG CAA TGA TCT CAA CAC G).

To measure ISG induction cells were infected with UV-treated Merlin CMV (MOI:2) for 6 h, followed by RNA extraction. Indicated genes were measured by relative quantification by qPCR using primers IFIT2 (F: ACT GCT GAA AGG GAG CTG AA, R: TGC ACA TTG TGG CTT TGA AT), IFIT3: (F: AGA AAT GAA AGG GCG AAG GT, R: ATG GCC TGC TTC AAA ACA TC), CXCL10: (F: TGG CAT TCA AGG AGT ACC TC, R: TTG TAG CAA TGA TCT CAA CAC G) with the same cycle parameters as above

### Virus binding

HFFs were infected at 4 °C for 1 h to allow virus binding. Cells were then washed in ice-cold phosphate-buffered saline (PBS) and total cell DNA was extracted and analysis by qPCR for viral genomes (using UL138 primers and conditions listed above).

### ELISA and CLIA

Serologic analysis was performed by either colorimetric ELISA or chemiluminescent immunoassay (CLIA). ELISA was performed on NUNC Maxisorp clear plates (Thermo) and CLIA on NUNC Maxisorp white plates (Thermo). Briefly, serum was diluted in PBS (1:50) and then incubated with peptide-coated 96-well plates (1 μg/mL in Carbonate-Bicarbonate buffer). Healthy seropositive and seronegative sera were used as controls. Anti-human IgG conjugated to HRP was used to detect CMV antibodies. Chromogenic substrate TMB (Thermo) was used for ELISA and SuperSignal ELISA pico Chemiluniscent substrate (Thermo) for CLIA assays.

### Cytometry

HFF cells were infected with IE2-GFP virus (MOI:5) cells and fixed with 2% PFA 3 days post infection. Cells were blocked (2% BSA) and stained with Live/Dead Aqua viability stain (Life Technologies, 1:1000 dilution), and an anti-AD-6 antibody at 50 μg/mL. Following 30 minutes of incubation, cells were washed and stained with PE-conjugated anti-rabbit IgG (L42018, Invitrogen; 1 μg/ml) and an APC-Cy7 conjugated HLA-A-C MHC class I antibody (clone W6/32Biolegend, 1:50 dilution) for 30 minutes, followed by washing. Infected cells were identified by MHC class I downregulation. Samples were acquired using an LSR Fortessa II, BD FASCDiva software and analysed with FlowJo v10.

### Statistical analysis

Statistical analysis was performed using GraphPad Prism software. Data were first evaluated for normal distribution and statistical tests were chosen accordingly. Specific tests are indicated in figure legends.

### Reporting summary

Further information on research design is available in the Nature Portfolio Reporting Summary linked to this article.

## Supplementary information

Supplementary Information

Reporting Summary

## Data Availability

All data shown in the manuscript is provided in the Source Data. Source data are provided with this paper.

## Source data

Source Data
