# Supplementary material for: The cytomegalovirus gB/MF59 vaccine candidate induces antibodies against an antigenic domain controlling cell-to-cell spread
Source: Nat Commun. 2023 Feb 23;14:1041. doi: 10.1038/s41467-023-36683-x (PMC9950427; doi:10.1038/s41467-023-36683-x)
Supplement: Supplementary file 1 — Supplementary Information [file 41467_2023_36683_MOESM1_ESM.pdf]

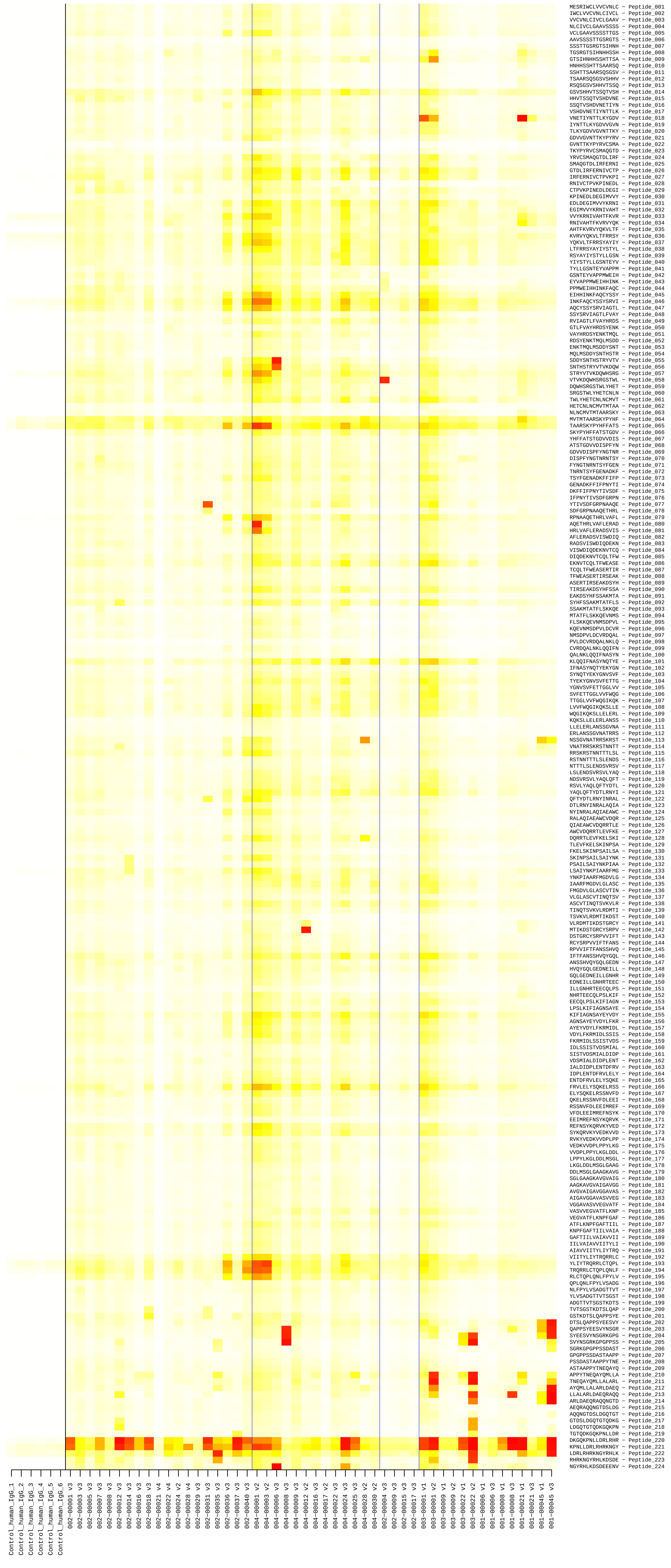
MESRIWCLVVCVNLC – Peptide_001
IWCLVVCVNLCIVCL – Peptide_002
VVCVNLCIVCLGAAV – Peptide_003
NLCIVCLGAAVSSSS – Peptide_004
VCLGAAVSSSSTTGS – Peptide_005
AAVSSSSTTGSRGTS – Peptide_006
SSSTTGSRGTSIHNH – Peptide_007
TGSRGTSIHNHHSSH – Peptide_008
GTSIHNHHSSHTTSA – Peptide_009
HNHHSSHTTSAARSQ – Peptide_010
SSHTTSAARSQSGSV – Peptide_011
TSAARSQSGSVSHHV – Peptide_012
RSQSGSVSHHVTSSQ – Peptide_013
GSVSHHVTSSQTVSH – Peptide_014
HHVTSSQTVSHDVNE – Peptide_015
SSQTVSHDVNETIYN – Peptide_016
VSHDVNETIYNTTLK – Peptide_017
VNETIYNTTLKYGDV – Peptide_018
IYNTTLKYGDVVGVN – Peptide_019
TLKYGDVVGVNTTKY – Peptide_020
GDVVGVNTTKYPYRV – Peptide_021
GVNTTKYPYRVCSMA – Peptide_022
TKYPYRVCSMAQGTD – Peptide_023
YRVCSMAQGTDLIRF – Peptide_024
SMAQGTDLIRFERNI – Peptide_025
GTDLIRFERNIVCTP – Peptide_026
IRFERNIVCTPVKPI – Peptide_027
RNIVCTPVKPINEDL – Peptide_028
CTPVKPINEDLDEGI – Peptide_029
KPINEDLDEGIMVVY – Peptide_030
EDLDEGIMVVYKRNI – Peptide_031
EGIMVVYKRNIVAHT – Peptide_032
VVYKRNIVAHTFKVR – Peptide_033
RNIVAHTFKVRVYQK – Peptide_034
AHTFKVRVYQKVLTF – Peptide_035
KVRVYQKVLTFRRSY – Peptide_036
YQKVLTFRRSYAYIY – Peptide_037
LTFRRSYAYIYSTYL – Peptide_038
RSYAYIYSTYLLGSN – Peptide_039
YIYSTYLLGSNTEYV – Peptide_040
TYLLGSNTEYVAPPM – Peptide_041
GSNTEYVAPPMWEIH – Peptide_042
EYVAPPMWEIHHINK – Peptide_043
PPMWEIHHINKFAQC – Peptide_044
EIHHINKFAQCYSSY – Peptide_045
INKFAQCYSSYSRVI – Peptide_046
AQCYSSYSRVIAGTL – Peptide_047
SSYSRVIAGTLFVAY – Peptide_048
RVIAGTLFVAYHRDS – Peptide_049
GTLFVAYHRDSYENK – Peptide_050
VAYHRDSYENKTMQL – Peptide_051
RDSYENKTMQLMSDD – Peptide_052
ENKTMQLMSDDYSNT – Peptide_053
MQLMSDDYSNTHSTR – Peptide_054
SDDYSNTHSTRYVTV – Peptide_055
SNTHSTRYVTVKDQW – Peptide_056
STRYVTVKDQWHSRG – Peptide_057
VTVKDQWHSRGSTWL – Peptide_058
DQWHSRGSTWLYHET – Peptide_059
SRGSTWLYHETCNLN – Peptide_060
TWLYHETCNLNCMVT – Peptide_061
HETCNLNCMVTMTAA – Peptide_062
NLNCMVTMTAARSKY – Peptide_063
MVTMTAARSKYPYHF – Peptide_064
TAARSKYPYHFFATS – Peptide_065
SKYPYHFFATSTGDV – Peptide_066
YHFFATSTGDVVDIS – Peptide_067
ATSTGDVVDISPFYN – Peptide_068
GDVVDISPFYNGTNR – Peptide_069
DISPFYNGTNRNTSY – Peptide_070
FYNGTNRNTSYFGEN – Peptide_071
TNRNTSYFGENADKF – Peptide_072
TSYFGENADKFFIFP – Peptide_073
GENADKFFIFPNYTI – Peptide_074
DKFFIFPNYTIVSDF – Peptide_075
IFPNYTIVSDFGRPN – Peptide_076
YTIVSDFGRPNAAQE – Peptide_077
SDFGRPNAAQETHRL – Peptide_078
RPNAAQETHRLVAFL – Peptide_079
AQETHRLVAFLERAD – Peptide_080
HRLVAFLERADSVIS – Peptide_081
AFLERADSVISWDIQ – Peptide_082
RADSVISWDIQDEKN – Peptide_083
VISWDIQDEKNVTCQ – Peptide_084
DIQDEKNVTCQLTFW – Peptide_085
EKNVTCQLTFWEASE – Peptide_086
TCQLTFWEASERTIR – Peptide_087
TFWEASERTIRSEAK – Peptide_088
ASERTIRSEAKDSYH – Peptide_089
TIRSEAKDSYHFSSA – Peptide_090
EAKDSYHFSSAKMTA – Peptide_091
SYHFSSAKMTATFLS – Peptide_092
SSAKMTATFLSKKQE – Peptide_093
MTATFLSKKQEVNMS – Peptide_094
FLSKKQEVNMSDPVL – Peptide_095
KQEVNMSDPVLDCVR – Peptide_096
NMSDPVLDCVRDQAL – Peptide_097
PVLDCVRDQALNKLQ – Peptide_098
CVRDQALNKLQQIFN – Peptide_099
QALNKLQQIFNASYN – Peptide_100
KLQQIFNASYNQTYE – Peptide_101
IFNASYNQTYEKYGN – Peptide_102
SYNQTYEKYGNVSVF – Peptide_103
TYEKYGNVSVFETTG – Peptide_104
YGNVSVFETTGGLVV – Peptide_105
SVFETTGGLVVFWQG – Peptide_106
TTGGLVVFWQGIKQK – Peptide_107
LVVFWQGIKQKSLLE – Peptide_108
WQGIKQKSLLELERL – Peptide_109
KQKSLLELERLANSS – Peptide_110
LLELERLANSSGVNA – Peptide_111
ERLANSSGVNATRRS – Peptide_112
NSSGVNATRRSKRST – Peptide_113
VNATRRSKRSTNNTT – Peptide_114
RRSKRSTNNTTTLSL – Peptide_115
RSTNNTTTLSLENDS – Peptide_116
NTTTLSLENDSVRSV – Peptide_117
LSLENDSVRSVLYAQ – Peptide_118
NDSVRSVLYAQLQFT – Peptide_119
RSVLYAQLQFTYDTL – Peptide_120
YAQLQFTYDTLRNYI – Peptide_121
QFTYDTLRNYINRAL – Peptide_122
DTLRNYINRALAQIA – Peptide_123
NYINRALAQIAEAWC – Peptide_124
RALAQIAEAWCVDQR – Peptide_125
QIAEAWCVDQRRTLE – Peptide_126
AWCVDQRRTLEVFKE – Peptide_127
DQRRTLEVFKELSKI – Peptide_128
TLEVFKELSKINPSA – Peptide_129
FKELSKINPSAILSA – Peptide_130
SKINPSAILSAIYNK – Peptide_131
PSAILSAIYNKPIAA – Peptide_132
LSAIYNKPIAARFMG – Peptide_133
YNKPIAARFMGDVLG – Peptide_134
IAARFMGDVLGLASC – Peptide_135
FMGDVLGLASCVTIN – Peptide_136
VLGLASCVTINQTSV – Peptide_137
ASCVTINQTSVKVLR – Peptide_138
TINQTSVKVLRDMTI – Peptide_139
TSVKVLRDMTIKDST – Peptide_140
VLRDMTIKDSTGRCY – Peptide_141
MTIKDSTGRCYSRPV – Peptide_142
DSTGRCYSRPVVIFT – Peptide_143
RCYSRPVVIFTFANS – Peptide_144
RPVVIFTFANSSHVQ – Peptide_145
IFTFANSSHVQYGQL – Peptide_146
ANSSHVQYGQLGEDN – Peptide_147
HVQYGQLGEDNEILL – Peptide_148
GQLGEDNEILLGNHR – Peptide_149
EDNEILLGNHRTEEC – Peptide_150
ILLGNHRTEECQLPS – Peptide_151
NHRTEECQLPSLKIF – Peptide_152
EECQLPSLKIFIAGN – Peptide_153
LPSLKIFIAGNSAYE – Peptide_154
KIFIAGNSAYEYVDY – Peptide_155
AGNSAYEYVDYLFKR – Peptide_156
AYEYVDYLFKRMIDL – Peptide_157
VDYLFKRMIDLSSIS – Peptide_158
FKRMIDLSSISTVDS – Peptide_159
IDLSSISTVDSMIAL – Peptide_160
SISTVDSMIALDIDP – Peptide_161
VDSMIALDIDPLENT – Peptide_162
IALDIDPLENTDFRV – Peptide_163
IDPLENTDFRVLELY – Peptide_164
ENTDFRVLELYSQKE – Peptide_165
FRVLELYSQKELRSS – Peptide_166
ELYSQKELRSSNVFD – Peptide_167
QKELRSSNVFDLEEI – Peptide_168
RSSNVFDLEEIMREF – Peptide_169
VFDLEEIMREFNSYK – Peptide_170
EEIMREFNSYKQRVK – Peptide_171
REFNSYKQRVKYVED – Peptide_172
SYKQRVKYVEDKVVD – Peptide_173
RVKYVEDKVVDPLPP – Peptide_174
VEDKVVDPLPPYLKG – Peptide_175
VVDPLPPYLKGLDDL – Peptide_176
LPPYLKGLDDLMSGL – Peptide_177
LKGLDDLMSGLGAAG – Peptide_178
DDLMSGLGAAGKAVG – Peptide_179
SGLGAAGKAVGVAIG – Peptide_180
AAGKAVGVAIGAVGG – Peptide_181
AVGVAIGAVGGAVAS – Peptide_182
AIGAVGGAVASVVEG – Peptide_183
VGGAVASVVEGVATF – Peptide_184
VASVVEGVATFLKNP – Peptide_185
VEGVATFLKNPFGAF – Peptide_186
ATFLKNPFGAFTIIL – Peptide_187
KNPFGAFTIILVAIA – Peptide_188
GAFTIILVAIAVVII – Peptide_189
IILVAIAVVIITYLI – Peptide_190
AIAVVIITYLIYTRQ – Peptide_191
VIITYLIYTRQRRLC – Peptide_192
YLIYTRQRRLCTQPL – Peptide_193
TRQRRLCTQPLQNLF – Peptide_194
RLCTQPLQNLFPYLV – Peptide_195
QPLQNLFPYLVSADG – Peptide_196
NLFPYLVSADGTTVT – Peptide_197
YLVSADGTTVTSGST – Peptide_198
ADGTTVTSGSTKDTS – Peptide_199
TVTSGSTKDTSLQAP – Peptide_200
GSTKDTSLQAPPSYE – Peptide_201
DTSLQAPPSYEESVY – Peptide_202
QAPPSYEESVYNSGR – Peptide_203
SYEESVYNSGRKGPG – Peptide_204
SVYNSGRKGPGPPSS – Peptide_205
SGRKGPGPPSSDAST – Peptide_206
GPGPPSSDASTAAPP – Peptide_207
PSSDASTAAPPYTNE – Peptide_208
ASTAAPPYTNEQAYQ – Peptide_209
APPYTNEQAYQMLLA – Peptide_210
TNEQAYQMLLALARL – Peptide_211
AYQMLLALARLDAEQ – Peptide_212
LLALARLDAEQRAQQ – Peptide_213
ARLDAEQRAQQNGTD – Peptide_214
AEQRAQQNGTDSLDG – Peptide_215
AQQNGTDSLDGQTGT – Peptide_216
GTDSLDGQTGTQDKG – Peptide_217
LDGQTGTQDKGQKPN – Peptide_218
TGTQDKGQKPNLLDR – Peptide_219
DKGQKPNLLDRLRHR – Peptide_220
KPNLLDRLRHRKNGY – Peptide_221
LDRLRHRKNGYRHLK – Peptide_222
RHRKNGYRHLKDSDE – Peptide_223
NGYRHLKDSDEEENV – Peptide_224
Control_human_IgG_1
Control_human_IgG_2
Control_human_IgG_3
Control_human_IgG_4
Control_human_IgG_5
Control_human_IgG_6
002-00001 v3
002-00003 v3
002-00005 v3
002-00007 v3
002-00008 v3
002-00012 v3
002-00014 v3
002-00016 v3
002-00018 v3
002-00021 v4
002-00022 v4
002-00024 v2
002-00028 v4
002-00029 v3
002-00031 v3
002-00035 v3
002-00036 v3
002-00037 v3
002-00040 v3
004-00001 v2
004-00003 v2
004-00006 v3
004-00008 v3
004-00009 v3
004-00012 v2
004-00016 v3
004-00017 v2
004-00022 v3
004-00024 v3
004-00025 v3
004-00028 v2
004-00030 v2
002-00004 v3
002-00009 v3
002-00015 v3
002-00017 v3
003-00001 v1
003-00001 v2
003-00009 v1
003-00009 v2
003-00022 v1
003-00022 v2
001-00006 v1
001-00006 v3
001-00008 v1
001-00008 v3
001-00021 v1
001-00021 v3
001-00045 v1
001-00045 v3

Figure S1 Breadth of the humoral response against linear epitopes of gB in vaccinated individuals. Representative heatmap representing antibody binding across the entirety of gB protein in sera from seronegative vaccine sera and seropositive control sera. White is no binding, Yellow is medium binding and red represents high binding. AD6 is located between peptide 162 and peptide 173 inclusive. For full dataset please supplementary dataset 1. 002-00001 to 002-00040 and 004-00001 to 004-00030 represent HCMV seronegative individuals receiving 1-2 doses of gB/MF59 vaccine. 002-0004 to 002-00017 represent HCMV seronegative patients receiving placebo. 003-00001 to 001-00045 represents HCMV seropositive patients receiving gB/MF59 vaccine. V1 refers to pre-vaccine sample and v2 onwards is post vaccine.

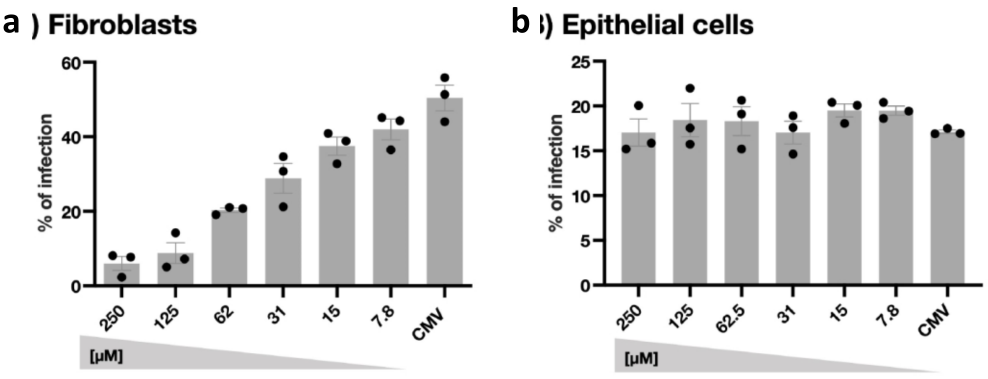

Figure S2 Full length AD-6 polypeptide blocks CMV infection in fibroblasts but not in epithelial cells. **a)** HFF cells were infected with Merlin (MOI:2) pre-incubated with AD-6 ($gB_{(648-697)}$) with concentrations ranging from 250μM to 3.9μM. **b)** ARPE-19 cells were infected with TB40e (MOI:3) pre-incubated with AD6 at concentrations ranging from 250μM to 7.8μM pre-treated with AD-6 prior to infection of ARPE-19. Infection was measured as a percentage of IE-positive 20h post infection. Bars represent mean + SEM of technical replicates and results are representative of 3 independent experiments.

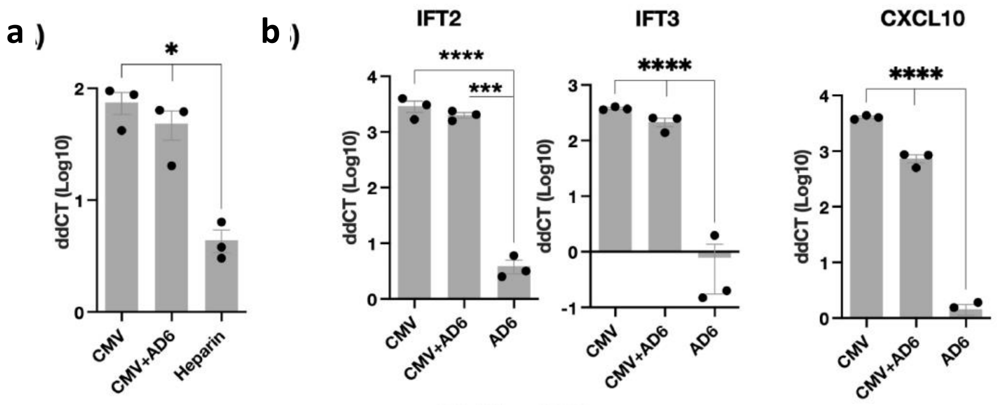

Figure S3 Impact of AD-6 polypeptide on post-attachment events during virus entry. **a)** Virus attachment to cells was measured in the presence or absence of AD-6 or Heparin. CMV Merlin strain (MOI:2), polypeptides (250µg/mL) or controls were inoculated on HFF for 90 minutes at 4°C and washed to remove unbound virions. Virion attachment was measured by the amount of viral DNA in the sample. Data expressed as UL138 Ct values normalized to housekeeping gene 18s. **b)** HFF cells infected with CMV UV treated (Merlin, MOI=2), AD-6 or CMV pre-treated with AD-6. 6hpi, RNA was isolated and mRNA levels of CXCL10, IFTI2, IFIT3 were measured by RT-qPCR (mean + SEM, n=3). Data expressed as fold change normalized to housekeeping gene 18s relative to mock treated cells. *P* values were calculated by a Kruskal-Wallis test with Dunn's multiple-comparison test where appropriate. ****, $P < 0.0001$; ***$P < 0.001$; *, $P < 0.01$; ns (nonsignificant), $P > 0.05$. For a and b the error bars represent 1 standard deviation from the mean.

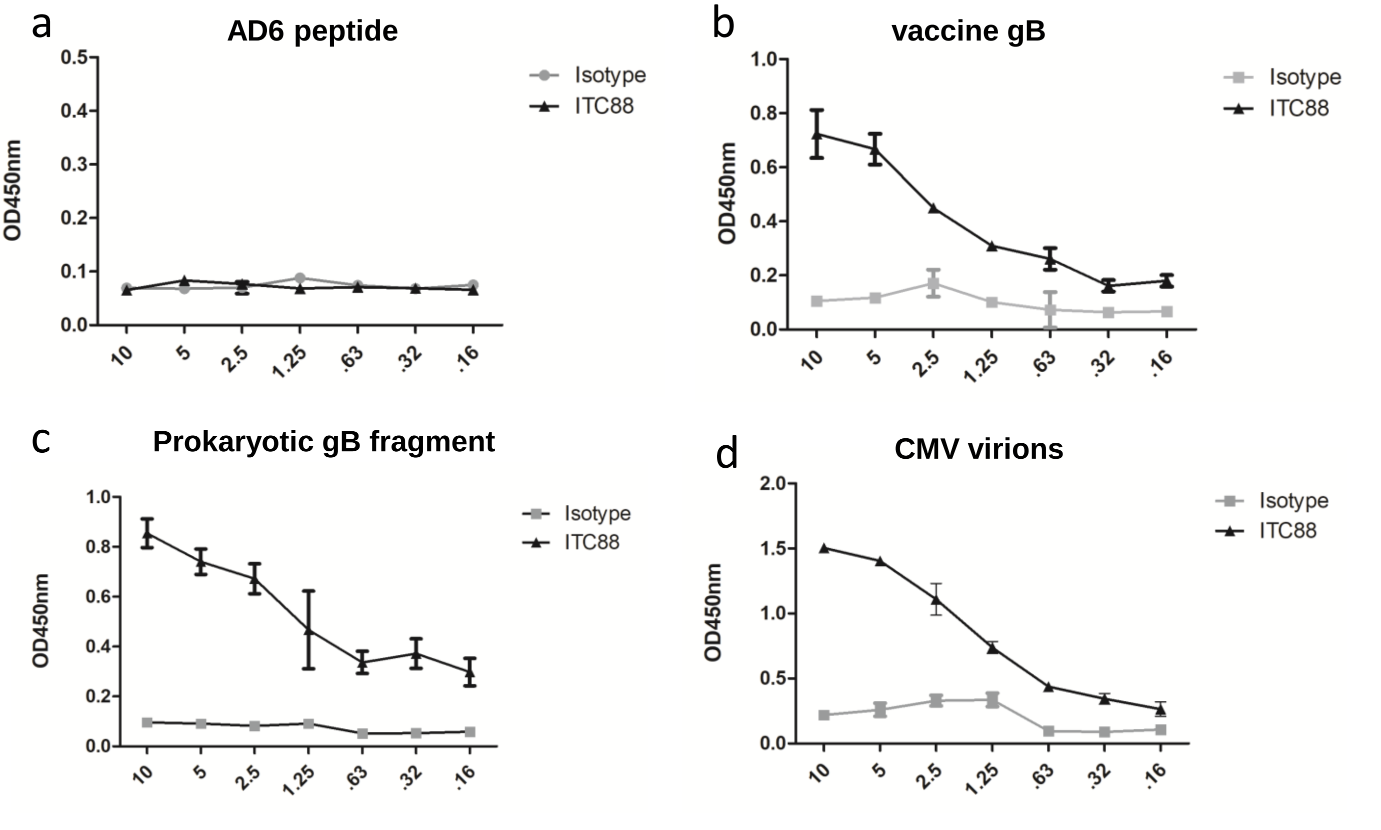

Figure S4 AD2 binding antibody, ITC88, binds recombinant gB, vaccine gB and virion gB a-d) An anti-AD2 monoclonal antibody (ITC88) or mouse isotype control were assayed by ELISA for binding to AD-6 polypeptide (a), vaccine gB (b), a commercial recombinant gB fragment produced in *E. coli (*c) or CMV virions (d). Antibody levels represented in function of absorbance (450nm) per concentration of primary antibody in mg/mL and is representative of 2 independent experiments. Each data point represents mean + SEM.

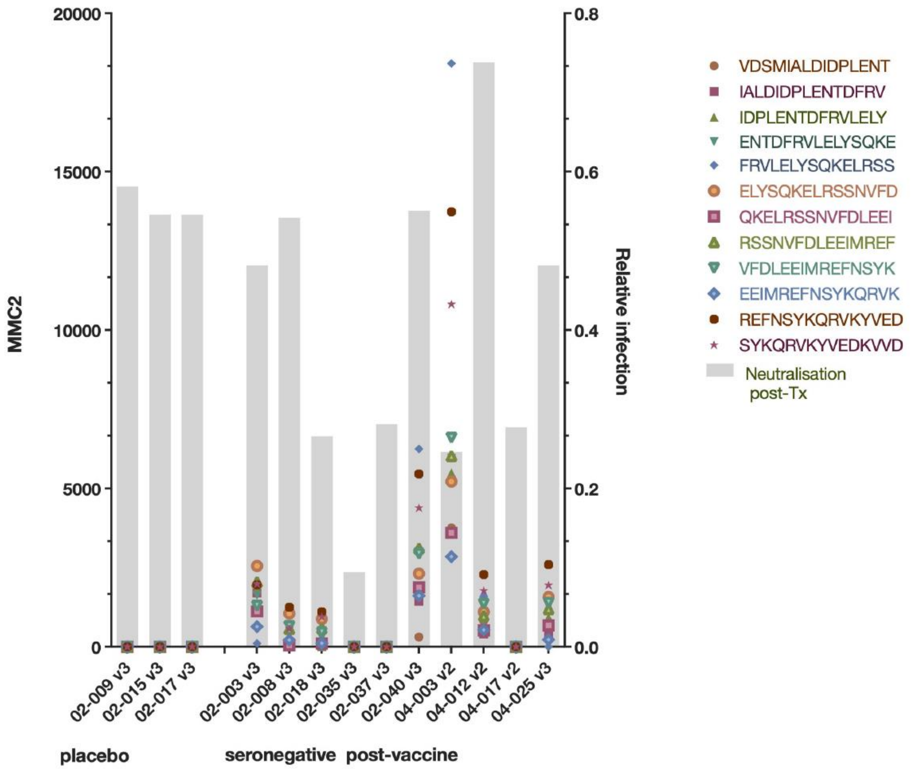

Figure S5 Presence of neutralising gB antibodies post-transplant does not correlate with AD-6 antibody responses pre-transplant. Anti-AD-6 IgG levels post-immunisation in seronegative vaccine recipients by array (left Y-axis – symbols) and capacity of post-transplant sera to neutralise CMV infection represented as reduction in the percentage of infected cells relative to mock-treated virus (right Y-axis; grey bars) were plotted together to compare neutralising activity with presence of antibody responses to epitopes within AD-6.

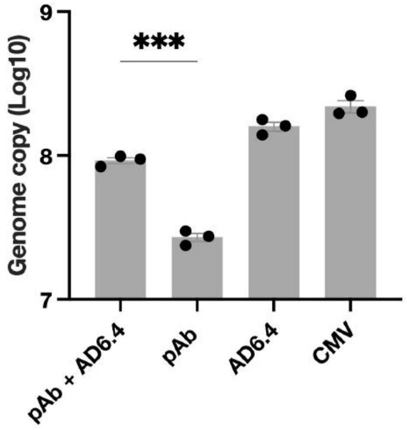

Figure S6 Anti-AD-6 antibodies reduce cell-associated spread in fibroblasts. HFFs were infected with BAC derived Merlin-IE2-GFP (MOI = 0.01). Then, at 1 dpi and at 5 dpi, anti-AD-6 antibody was added at the indicated concentrations except for some conditions the anti-AD-6 antibody was pre-adsorbed with shorter peptide spanning the C-terminus of AD-6, herein named AD-6.4 ($gB_{(685\text{-}699),}$ Towne) at a 10x molar excess prior to addition to infected cells 1 dpi and at 5 dpi. Then, at 10 dpi. total DNA was harvested, and CMV genome copies per 10^6 cells assessed by qPCR ($n = 2$). P values were calculated by a two tailed unpaired t test. ***, $P < 0.000194$. The error bars represent 1 standard error from the mean.
