# Supplementary material for: The cytomegalovirus gB/MF59 vaccine candidate induces antibodies against an antigenic domain controlling cell-to-cell spread
Source: Nat Commun. 2023 Feb 23;14:1041. doi: 10.1038/s41467-023-36683-x (PMC9950427; doi:10.1038/s41467-023-36683-x)
Supplement: Supplementary file 2 — Reporting Summary [file 41467_2023_36683_MOESM2_ESM.pdf]

nature portfolio

Corresponding author(s): Reeves

Last updated by author(s): Feb 1, 2023

Reporting Summary

Nature Portfolio wishes to improve the reproducibility of the work that we publish. This form provides structure for consistency and transparency in reporting. For further information on Nature Portfolio policies, see our Editorial Policies and the Editorial Policy Checklist.

Statistics

For all statistical analyses, confirm that the following items are present in the figure legend, table legend, main text, or Methods section.

n/a	Confirmed	
☐	☒	The exact sample size (*n*) for each experimental group/condition, given as a discrete number and unit of measurement
☐	☒	A statement on whether measurements were taken from distinct samples or whether the same sample was measured repeatedly
☐	☒	The statistical test(s) used AND whether they are one- or two-sided<br>*Only common tests should be described solely by name; describe more complex techniques in the Methods section.*
☒	☐	A description of all covariates tested
☒	☐	A description of any assumptions or corrections, such as tests of normality and adjustment for multiple comparisons
☒	☐	A full description of the statistical parameters including central tendency (e.g. means) or other basic estimates (e.g. regression coefficient) AND variation (e.g. standard deviation) or associated estimates of uncertainty (e.g. confidence intervals)
☐	☒	For null hypothesis testing, the test statistic (e.g. *F*, *t*, *r*) with confidence intervals, effect sizes, degrees of freedom and *P* value noted<br>*Give P values as exact values whenever suitable.*
☒	☐	For Bayesian analysis, information on the choice of priors and Markov chain Monte Carlo settings
☒	☐	For hierarchical and complex designs, identification of the appropriate level for tests and full reporting of outcomes
☒	☐	Estimates of effect sizes (e.g. Cohen's *d*, Pearson's *r*), indicating how they were calculated

Our web collection on statistics for biologists contains articles on many of the points above.

Software and code

Policy information about availability of computer code

Data collection

In order to perform sequence conservation analysis, a selection of full-length amino acid sequences were downloaded from GenBank (search terms: "Human betaherpesvirus 5 glycoprotein B ", filtered by 880-920 amino acid sequence length). A multiple sequence alignment was assembled using MAFFT online server. (https://doi.org/10.1093/bib/bbx108). Sequences from synthetic strains were removed. Towne strain (GenBank: ABQ23592.1) was used as a reference.

To study AD6 location in the gB structure the region corresponding to AD6 (aa 648 – 697, Towne) were highlighted in each side chain of the Homotrimer of Towne Glycoprotein B structure in the prefusion (PDB: 7KDP) and postfusion (PDB: 7KDD) conformation. Structure files were collected from https://www.rcsb.org/. Visualisation was performed using 3-D structure viewer Geneious Prime® 2021.2.2

Percentage infection was assessed by automated fluorescence microscopy and image recognition Hermes WiScan (IDEA Bio-Medical) instruments and processed by MetaMorph software (Molecular devices).

Phenotypic analysis was carried out with data acquired on an LSRFortessa II (BD Biosciences)

Data analysis

Data analysis was performed using GraphPad Prism Software. flow cytometry data was analysed using FlowJo version 10.5.3

For manuscripts utilizing custom algorithms or software that are central to the research but not yet described in published literature, software must be made available to editors and reviewers. We strongly encourage code deposition in a community repository (e.g. GitHub). See the Nature Portfolio guidelines for submitting code & software for further information.

Data

Policy information about availability of data

All manuscripts must include a data availability statement. This statement should provide the following information, where applicable:

- Accession codes, unique identifiers, or web links for publicly available datasets
- A description of any restrictions on data availability
- For clinical datasets or third party data, please ensure that the statement adheres to our policy

All data shown in the manuscript is provided in the source data file. Requests for the raw data (e.g. Fig. S1) should be directed to the corresponding author and will be made available on request.

Field-specific reporting

Please select the one below that is the best fit for your research. If you are not sure, read the appropriate sections before making your selection.

☒ Life sciences ☐ Behavioural & social sciences ☐ Ecological, evolutionary & environmental sciences

For a reference copy of the document with all sections, see nature.com/documents/nr-reporting-summary-flat.pdf

Life sciences study design

All studies must disclose on these points even when the disclosure is negative.

Sample size	sample sizes were n=3 except for the analysis of clinical samples which was dictated by number of samples available or where otherwise stated
Data exclusions	No data was excluded
Replication	investigations were repeated multiple times on separate days with different cells etc. In a number of instances multiple investigators performed repeats of the same experiments.
Randomization	Samples were not randomised as study was investigating the activity of a known reagent. Sera from the original study was derived from a placebo controlled double blind trial
Blinding	no blinding was performed in these experiments - see comment above about sera from clinical trial

Reporting for specific materials, systems and methods

We require information from authors about some types of materials, experimental systems and methods used in many studies. Here, indicate whether each material, system or method listed is relevant to your study. If you are not sure if a list item applies to your research, read the appropriate section before selecting a response.

Materials & experimental systems

n/a	Involved in the study	
☐	☒	Antibodies
☐	☒	Eukaryotic cell lines
☒	☐	Palaeontology and archaeology
☒	☐	Animals and other organisms
☐	☒	Human research participants
☐	☒	Clinical data
☒	☐	Dual use research of concern

Methods

n/a	Involved in the study	
☒	☐	ChIP-seq
☐	☒	Flow cytometry
☒	☐	MRI-based neuroimaging

Antibodies

Antibodies used	mouse anti-IE (MAB8131; clone 6F8.2; Merck Millipore; 1:2,000 dilution)<br>Rabbit anti-AD6 antibody (range of concentrations)<br>mouse HLA class I APCcy7 conjugated antibody (clone W6/32, Biolegend, 1:50 dilution)<br>goat anti-rabbit IgG PE - (L42018, Invitrogen, 1ug/ml)
Validation	Each commercial antibody is used extensively in our lab and was used based on website verification and our own in house analyses.<br>The rabbit AD-6 antibody was validated in the manuscript data presented.

Eukaryotic cell lines

Policy information about cell lines

Cell line source(s)	ATCC
Authentication	ATCC validation - trusted source
Mycoplasma contamination	We routinely check for mycoplasma in the lab and the cells were mycoplasma free at time of analyses
Commonly misidentified lines (See ICLAC register)	*Name any commonly misidentified cell lines used in the study and provide a rationale for their use.*

Human research participants

Policy information about studies involving human research participants

Population characteristics	*Describe the covariate-relevant population characteristics of the human research participants (e.g. age, gender, genotypic information, past and current diagnosis and treatment categories). If you filled out the behavioural & social sciences study design questions and have nothing to add here, write "See above."*
Recruitment	*Describe how participants were recruited. Outline any potential self-selection bias or other biases that may be present and how these are likely to impact results.*
Ethics oversight	*Identify the organization(s) that approved the study protocol.*

Note that full information on the approval of the study protocol must also be provided in the manuscript.

Clinical data

Policy information about clinical studies

All manuscripts should comply with the ICMJE guidelines for publication of clinical research and a completed CONSORT checklist must be included with all submissions.

Clinical trial registration	This study uses samples from a prior clinical trial NCT00299260
Study protocol	*Note where the full trial protocol can be accessed OR if not available, explain why.*
Data collection	*Describe the settings and locales of data collection, noting the time periods of recruitment and data collection.*
Outcomes	*Describe how you pre-defined primary and secondary outcome measures and how you assessed these measures.*

Flow Cytometry

Plots

Confirm that:

- [x] The axis labels state the marker and fluorochrome used (e.g. CD4-FITC).
- [x] The axis scales are clearly visible. Include numbers along axes only for bottom left plot of group (a 'group' is an analysis of identical markers).
- [x] All plots are contour plots with outliers or pseudocolor plots.
- [ ] A numerical value for number of cells or percentage (with statistics) is provided.

Methodology

Sample preparation	Infected and uninfected fibroblasts were fixed with 2% PFA and then stained with anti-AD6 antibody (50ug/ml) or rabbit IgG control for 30 minutes, washed and then incubated with either anti-rabbit PE (1ug/ml) and HLA-A,B,C APC-cy7 antibody (1:50 dilution) for 30 minutes and then washed and left on ice until analysis.
Instrument	LSR Fortessa II
Software	BD FACSDiva Software
Cell population abundance	These experiments were performed on homogeneous fibroblasts and thus account for 100% of the population.
Gating strategy	gates were set using uninfected cells for AD6 antibody which is shown in main figure

- [ ] Tick this box to confirm that a figure exemplifying the gating strategy is provided in the Supplementary Information.
